# Quaternary Ammonium Leucine-Based Surfactants: The Effect of a Benzyl Group on Physicochemical Properties and Antimicrobial Activity

**DOI:** 10.3390/pharmaceutics11060287

**Published:** 2019-06-19

**Authors:** Diego Romano Perinelli, Dezemona Petrelli, Luca Agostino Vitali, Giulia Bonacucina, Marco Cespi, Driton Vllasaliu, Gianfabio Giorgioni, Giovanni Filippo Palmieri

**Affiliations:** 1 School of Pharmacy, University of Camerino, 62032 Camerino, Italy; luca.vitali@unicam.it (L.A.V.); giulia.bonacucina@unicam.it (G.B.); marco.cespi@unicam.it (M.C.); gianfabio.giorgioni@unicam.it (G.G.); gianfilippo.palmieri@unicam.it (G.F.P.); 2 School of Biosciences and Veterinary Medicine, University of Camerino, 62032 Camerino, Italy; dezemona.petrelli@unicam.it; 3 School of Cancer and Pharmaceutical Sciences, Faculty of Life Sciences and Medicine, King’s College London, London SE1 9NH, UK; driton.vllasaliu@kcl.ac.uk

**Keywords:** cationic surfactants, amino acid, surface tension, antibacterial activity, toxicological studies

## Abstract

Quaternary ammonium amphiphiles are a class of compounds with a wide range of commercial and industrial uses. In the pharmaceutical field, the most common quaternary ammonium surfactant is benzalkonium chloride (BAC), which is employed as a preservative in several topical formulations for ocular, skin, or nasal application. Despite the broad antimicrobial activity against Gram-positive and Gram-negative bacteria, as well as fungi and small enveloped viruses, safety concerns regarding its irritant and cytotoxic effect on epithelial cells still remain. In this work, quaternary ammonium derivatives of leucine esters (C10, C12 and C14) were synthesised as BAC analogues. These cationic surfactants were characterised in terms of critical micelle concentration (CMC, by tensiometry), cytotoxicity (MTS and LDH assays on the Caco-2 and Calu-3 cell lines) and antimicrobial activity on the bacterial species *Staphylococcus aureus* and *Enterococcus faecalis* among the Gram-positives, *Escherichia coli* and *Pseudomonas aeruginosa* among the Gram-negatives and the yeast *Candida albicans*. They showed satisfactory surface-active properties, and a cytotoxic effect that was dependent on the length of the hydrophobic chain. Lower minimum inhibiting concentration (MIC) values were calculated for C14-derivatives, which were comparable to those calculated for BAC toward Gram-positive bacteria and slightly higher for Gram-negative bacteria and *C. albicans*. Thus, the synthesised leucine-based quaternary ammonium cationic surfactants can potentially find application as promising surface-active compounds with antimicrobial activity.

## 1. Introduction

Amino-acid-based surfactants are a promising class of amphiphiles obtained by the condensation of an amino acid as a polar head and one or two acyl/alkyl chains as hydrophobic tails [1]. These materials are occasionally referred to as “green surfactants”, since they are environmentally friendly and can be produced according to the principles of green chemistry starting from renewable materials [2]. The physicochemical properties of these amphiphiles can be tuned by varying the nature of the amino acid, the number of acyl/alkyl chains and the type of linkage (amide, ester or alkyl) between the amino acid and the hydrophobic portion, giving a large variety of linear (single-chain), double-chain or dimeric (gemini) surfactants [3]. According to the site of the introduction of the hydrophobic tail on the amino acid functional groups (carboxylic or amine) and the charge of the amino acid side chain, anionic, cationic and zwitterionic amphiphiles can be obtained. Both anionic and cationic amino-acid-based surfactants have been widely investigated and proposed in pharmaceutical and cosmetics formulations as alternatives to commercial surfactants due to their favourable physicochemical properties (e.g., foamability and emulsification) and toxicological profiles [4]. Generally, they are considered to be biocompatible and biodegradable, with studies having confirmed their low toxicity on selected cell lines [5,6,7]. Cationic-based amphiphiles of this class of materials have been explored extensively for their antimicrobial activity toward both bacteria and fungi [8]. To this end, different arginine-, lysine- or histidine-based single chain or dimeric surfactants have been developed [9,10,11]. All these compounds have exhibited a wide spectrum of action and showed a better inhibitory activity against Gram-positive bacteria than Gram-negative bacteria and fungi, which depends mainly on the length of the hydrocarbon chain in addition to the nature of the amino acid residue. The best activity was observed for C12/C14 derivatives as a result of an optimal hydrophobic/hydrophilic balance that enhanced the interaction and penetration of the surfactant inside the membrane of the micro-organism [9,12]. Quaternary ammonium cationic amphiphiles can also be obtained from amino acids via alkylation of the amine group. These compounds have not been fully investigated in terms of their physicochemical and biological properties. Indeed, the formation of quaternary ammonium salts is an established strategy for obtaining antimicrobial compounds that has already been explored for other new classes of amphiphiles [13]. In a previous paper, we synthesised and characterised the surface activity, cytotoxicity, and antimicrobial properties of two series of linear quaternary ammonium compounds, based on the amino acid leucine and methionine and with different hydrophobic chain lengths (C10, C12 and C14). These amphiphiles, especially derivatives with a C12 and C14 hydrocarbon chain, showed a satisfactory cytotoxicity and an antibacterial profile that was comparable to benzalkonium chloride (BAC), with higher activity observed against Gram-positive bacteria [14]. In the present work, we report the synthesis of the same series of leucine-based surfactants by introducing an *N*-benzyl group, which is a common substituent of commercial quaternary ammonium compounds (e.g., benzalkonium chloride). Then, we investigated the surface properties, cytotoxicity and antimicrobial activity of these novel materials.

## 2. Materials and Methods

### 2.1. Materials

l-leucine (LEU), 1-decanol, *p*-toluenesulfonic acid (*p*-TSA), toluene anhydrous (99.8%), benzaldehyde, sodium cyanoborohydride and iodomethane purum ≥99.0% were purchased from Sigma-Aldrich (Milan, Italy). 1-dodecanol and 1-tetradecanol were obtained from DPI (Lancaster, SC, USA) and sodium carbonate and potassium carbonate from Carlo Erba (Milan, Italy). All used solvents (acetone, ethyl acetate, diethyl ether and methanol) were of analytical grade. Benzalkonium chloride (BAC, purity ≥95.0%), composed of benzyldimethyldodecylammonium chloride (~70%) and benzyldimethyltetradecylammonium chloride (~30%) (from the HPLC area% according to the manufacturer’s specifications, average molecular weight 348.41 Da) was purchased from Sigma-Aldrich (Milan, Italy). Dulbecco’s Modified Eagle Medium (DMEM), foetal bovine serum (FBS), antibiotic/antimycotic solution (10–12,000 U/mL penicillin, 10–12 mg/mL streptomycin, 25–30 μg/mL amphotericin B) and trypsin–EDTA solution (2.5 mg/mL trypsin, 0.2 mg/mL EDTA) were obtained from Sigma-Aldrich (Poole, UK). The MTS reagent 3-(4,5-dimethylthiazol-2-yl)-5(3-carboxymethonyphenol)-2-(4-sulphophenyl)-2H-tetrazolium (CellTiter96^®^ AQueous One Solution Cell Proliferation Assay) was purchased from Promega (Madison, WI, USA). A Pierce^TM^ LDH assay kit was purchased from ThermoScientific (Waltham, MA, USA). Tissue culture flasks (75 cm^3^ with ventilated caps) were purchased from Costar. Ultrapure water was obtained with a laboratory deionizer (Osmo lab UPW2, Gamma 3, (Castelverde, Italy).

### 2.2. Methods

#### 2.2.1. Synthesis and Chemical Characterisation of Benzyl Quaternary Ammonium Leucine-Based Surfactants

**Scheme 1:**
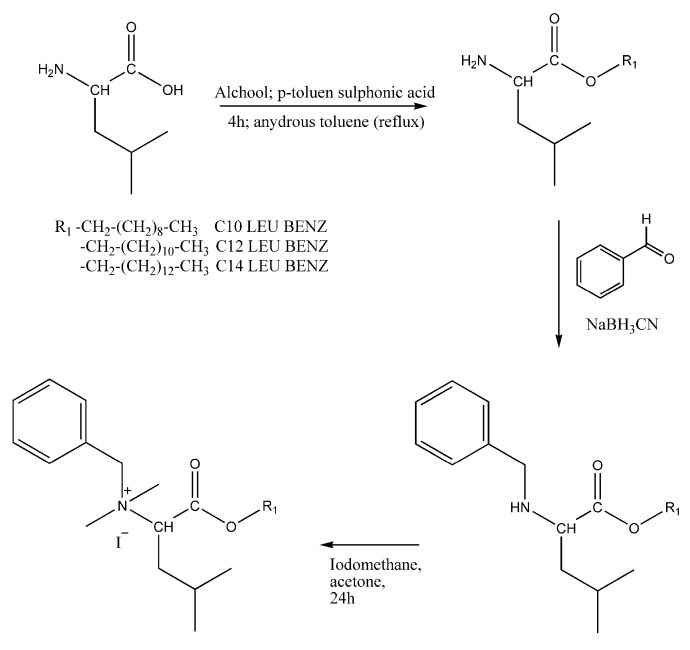
Synthetic approach to the preparation of benzyl quaternary ammonium leucine-based surfactants.

The benzyldimethyl ammonium salts of l-leucine, esterified with fatty acids of different lengths (C10, C12 and C14), were prepared using three steps. The esterification of leucine with the corresponding alcohol gave the *O*-acyl leucine derivatives. Subsequently, the benzyl-substituted secondary amine was prepared by reductive amination (imine formation and reduction). Finally, the quaternary ammonium salts were obtained by methylation of the *N*-benzyl secondary amine of the *O*-acyl amino acid. The overall synthetic approach is reported in Scheme 1.

##### Synthesis of *O*-Acyl Leucine Derivatives

In an anhydrous flask, 2 g of l-leucine were dispersed in anhydrous toluene (40 mL), and then the appropriate alcohol (1-decanol, 1-dodecanol, 1-tetradecanol; molar ratio 1:1.1) was added. To this mixture, *p*-toluenesulfonic acid (molar ratio 1:1.2) was gradually added and the reaction was refluxed for 4 h using a Dean–Stark apparatus. Afterwards, the toluene was evaporated under vacuum, and an oil as a crude product was obtained. The oily crude residue was dissolved in ethyl acetate and the excess *p*-toluenesulfonic acid was extracted using Na_2_CO_3_. The organic phase was collected and then dehydrated with anhydrous sodium sulfate. After filtration and solvent evaporation under vacuum, the compound was obtained as an oil [15,16].

##### Synthesis of *N*-Benzyl-*O*-Acyl Leucine Derivatives

To a solution of *O*-acyl leucine derivative (C10, C12, or C14) in methanol, NaCNBH_3_ (molar ratio 1:0.91) and benzaldehyde (molar ratio 1:1.1) were added and pH-adjusted to 6.0 with acetic acid. The mixture was stirred for 24 h at room temperature and, then, hydrolysed with K_2_CO_3_ (40% *w/v* in water). Subsequently, the aqueous phase was extracted using diethyl ether. The organic phase was collected, dried with anhydrous sodium sulfate, filtered and the solvent evaporated under vacuum, yielding a colourless oil [17].

##### Synthesis of *N*,*N*,*N*-Benzyldimethyl-*O*-Acyl Leucine Derivatives

K_2_CO_3_ (molar ratio 1:3) and CH_3_I (molar ratio 1:3) were added dropwise to a solution of the synthesised *N*-benzyl-*O*-acyl leucines in acetone (100 mL). The mixture was stirred for 24 h at room temperature, then cooled down to 4 °C and filtered to remove K_2_CO_3_. After evaporation of the organic solvent under vacuum, the quaternary ammonium salt was separated from the crude product by precipitation in ethyl ether. Finally, the crude solid was purified by repeated crystallisation from ethanol [18].

Chemical identification of the surfactants was performed by ^1^H-NMR (Varian EM-400 MHz spectrometer, Palo Alto, CA, USA) using deuterated DMSO as a solvent. Chemical structures, relative abbreviations and identification of the ^1^H-NMR signals for all synthesised surfactants are reported in the Supplementary Materials (Table S1).

#### 2.2.2. Surface Tension Measurements

Different concentrations for each surfactant were prepared by dissolving the compounds in ultrapure water. Their ability to decrease air–water surface tension was studied at 25 °C using a DCA-100 tensiometer (First Ten Angstrom, Portsmouth, VA, USA) according to the De Nouy ring method. Each recorded surface tension value was the mean of three consecutive measurements. The ‘breakpoint’ of the plot of surface tension versus concentration of surfactant was used to calculate the critical micelle concentration (CMC, i.e., the minimum concentration at which surfactants aggregate to form micelles) and the surface tension at CMC (γ_CMC_). The “breakpoint” is the point of intersection of the two lines fitting the experimental data by a linear regression model (Prism version 5.0, GraphPad Inc., San Diego, CA, USA). Tensiometric measurements allowed for the calculation of an additional two parameters related to the surface behaviour of surfactants. The first is the ‘surface excess concentration’, *Γ*_max_, which is a measure of how many surfactant molecules are adsorbed at the air–water interface, which was calculated using the Gibbs equation:

(1) $$\Gamma_{\max}=\frac{1}{2.303\;nRT}\;\left(\frac{\delta \gamma }{\delta \log C}\right)$$

where *R* is the gas constant (8.314 J/mol K); *T* is the absolute temperature (K); *n* is equal to 1 for single chain ionic surfactants displaying only one ionisable group; and dγ/d log *C* can be calculated from the slope of the straight section before the CMC in the plot of surface tension versus concentration of the surfactant.

The other parameter is the area occupied by a single molecule of surfactant at the water–air interface (*A_s_*), and is derived from *Γ*_max_ through the equation:

(2) $$A_{\min}=\;\frac{10^{18}}{N\Gamma_{\max}}.$$

All analyses were performed in triplicate.

#### 2.2.3. Cytotoxicity Studies (MTS and LDH Assays) and Haemolytic Assay

Colorectal-carcinoma-derived epithelial Caco-2 cells (European Collection of Cell Cultures) and airway epithelial Calu-3 cells (American Type Culture Collection; ATCC) were cultured in DMEM supplemented with 10% of FBS and 1% of an antibiotic–antimycotic mixture. Erythrocytes were collected from mice blood. The cytotoxicity of the *N*-benzyl quaternary ammonium surfactants derived from leucine in comparison to the commercial BAC was studied by performing two cytotoxicity assays (MTS and LDH as indicators of metabolic activity and membrane integrity, respectively) and by a haemolytic assay. The assays were conducted as reported in our previous publications [5,14]. EC_50_ values from the MTS assay (concentration of surfactant that causes 50% cell death) and the LDH assay (concentration of surfactants that causes 50% LDH release from cells), as well as HC_50_ values from the haemolytic assay (concentration of surfactants that causes 50% haemoglobin release from erythrocytes) were calculated using a nonlinear regression model (Prism version 5.0, GraphPad Inc., San Diego, CA, USA) as follows:

(3) $$Y=\mathrm{BOTTOM}+\frac{\mathrm{Top}-\mathrm{Bottom}}{1+10^{\left(\mathrm{LogEC}50-x\right)\;\mathrm{Hill}\;\mathrm{Slope}}}$$

where Top and Bottom are plateaux in the units of the *Y* axis.

#### 2.2.4. Antimicrobial Assay and MIC Determination

The antimicrobial activity was evaluated against the bacterial and fungal species listed in Table 1. Bacterial strains were cultured overnight at 35–36 °C in blood agar plates. Minimum inhibitory concentrations (MICs) were determined according to previous reports [14] and following protocols defined by the European Committee on Antimicrobial Susceptibility Testing (EUCAST). MICs against *Candida albicans* were determined using a similar protocol with some adaptations to the yeast growth and requirements. All tests were done in triplicate.

## 3. Results

### 3.1. Tensiometric Analysis

**Figure 1:**
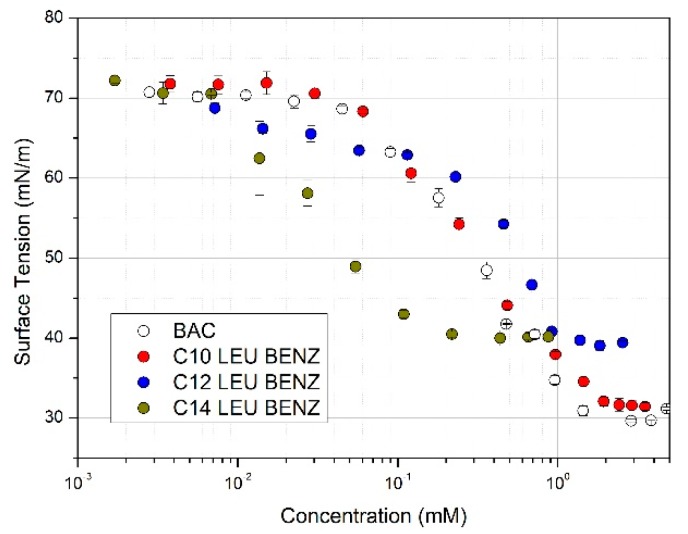
Surface tension versus concentration plot for the synthesised quaternary ammonium leucine surfactants (C10 LEU BENZ, C12 LEU BENZ and C14 LEU BENZ) surfactants in comparison to benzalkonium chloride (BAC).

Figure 1 shows the decrease in surface tension (mN/m) as a function of concentration for the synthesised quaternary ammonium leucine surfactants and BAC as a comparison. The calculated CMC values were influenced by the hydrophobicity, which is the length of the hydrocarbon chain. Particularly, C12 LEU BENZ displayed a CMC comparable to that of BAC (around 1 mM). All the derivatives had good surface properties, as demonstrated by γ_CMC_ and *Γ*_max_ (Table 2). For C10 LEU BENZ, γ_CMC_ was around 30 mN/m, similarly to BAC, but it increased up to 40 mN/m for the derivatives with a longer hydrocarbon chain (C12 LEU BENZ and C14 LEU BENZ). *Γ*_max_ values, indicating the tendency of the molecules to adsorb at the air–water interface, were in the order of 10^6^ (mol/m^2^) and comparable to BAC.

### 3.2. Cytotoxicity Studies and the Haemolitic Assay

**Figure 2:**
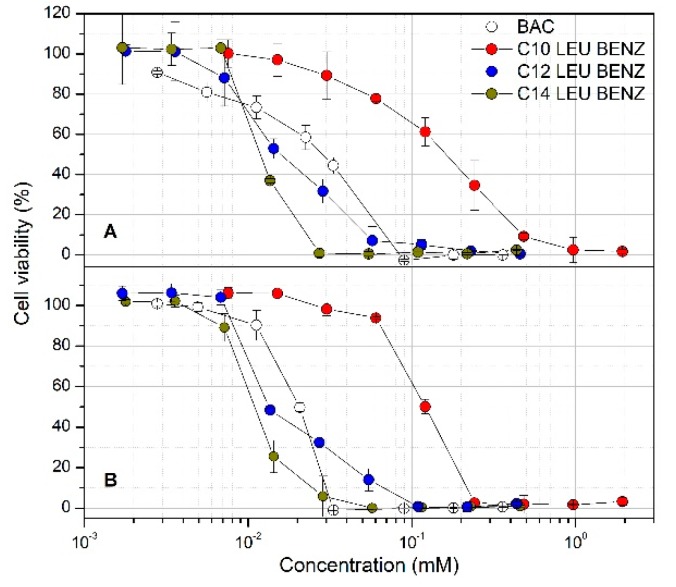
Cell viability (%) of Caco-2 (intestinal epithelial) (**A**) and Calu-3 (airway epithelial) (**B**) cell lines after exposure to different concentrations of the quaternary ammonium leucine surfactants (C10 LEU BENZ, C12 LEU BENZ and C14 LEU BENZ) and the reference compound BAC. Dots represent the mean and bars the standard error (*n* = 4).

**Figure 3:**
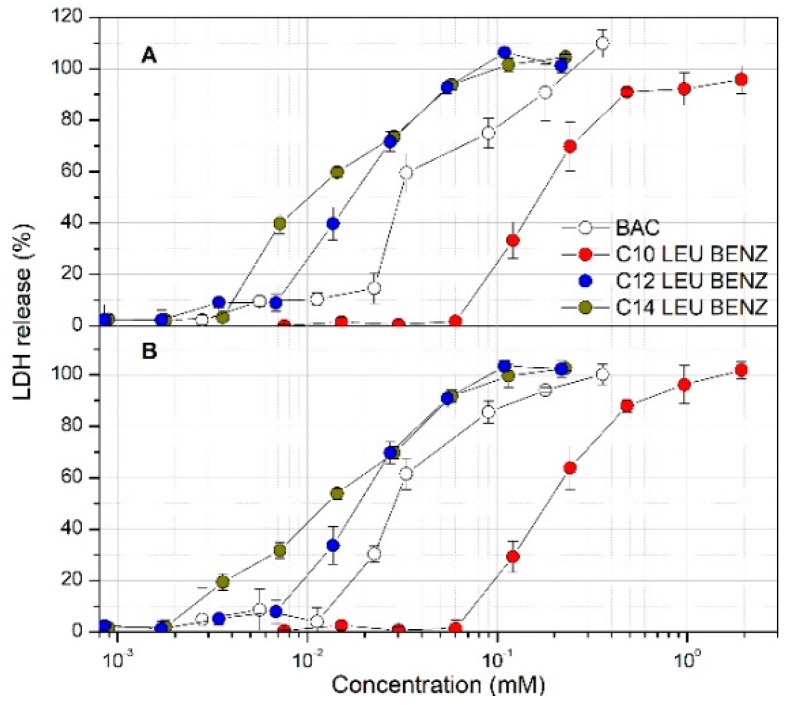
LDH release (%) of Caco-2 (intestinal epithelial) (**A**) and Calu-3 (airway epithelial) (**B**) cell lines after exposure to different concentrations of the quaternary ammonium leucine surfactants (C10 LEU BENZ, C12 LEU BENZ and C14 LEU BENZ) and the reference compound BAC. Dots represent the mean and bars the standard error (*n* = 4).

Cytotoxicity on the two selected cell lines, namely intestinal epithelial Caco-2 and airway epithelial Calu-3 (Figure 2 and Figure 3, respectively) showed a clear dependence on the length of the hydrocarbon chain for the synthesised surfactants as demonstrated from the calculated EC_50_ values, which decreased from C10 to C14 derivatives. These values were at least 10-fold lower than the CMC, and, for the C12 and C14 derivatives, they were comparable to BAC (Table 2). No notable differences were found between the two selected cell lines in terms of EC_50_ with both cytotoxicity assays (MTS and LDH assays) for all analysed surfactants.

**Figure 4:**
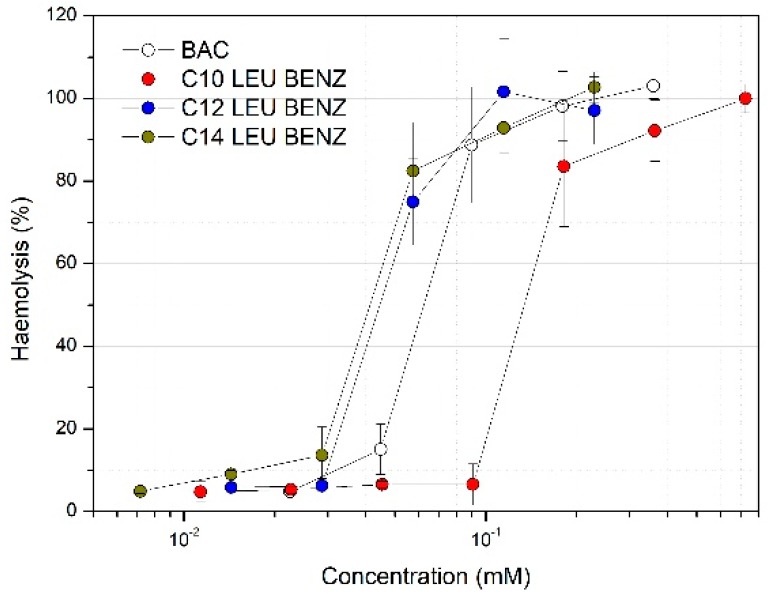
Haemolysis (%) after exposure of the quaternary ammonium leucine surfactants (C10 LEU BENZ, C12 LEU BENZ and C14 LEU BENZ) and the reference compound BAC. Dots represent the mean and bars the standard deviation (*n* = 3).

The haemolytic activity of different concentrations of the synthesised ammonium quaternary surfactants was tested in comparison to BAC (Figure 4). The results obtained in this experiment mirror the trends observed in the cytotoxicity studies, with the C10 LEU BENZ surfactant showing a lower haemolytic activity than the C12 and C14 derivatives. The calculated HC_50_ values are reported in Table 3.

### 3.3. Antimicrobial Assay

The results from the antimicrobial assay in terms of minimum inhibitory concentration (MIC) are reported in Table 4. Similarly to BAC, all synthesised quaternary ammonium surfactants were more effective against Gram-positive bacterial species and the fungus *C. albicans* than Gram-negative bacteria. The least susceptible bacteria was the Gram-negative *Pseudomonas aeruginosa*. C14 derivatives displayed the highest activity against all micro-organisms, especially against Gram-positive bacteria, and showed MIC values comparable to BAC.

**Figure 5:**
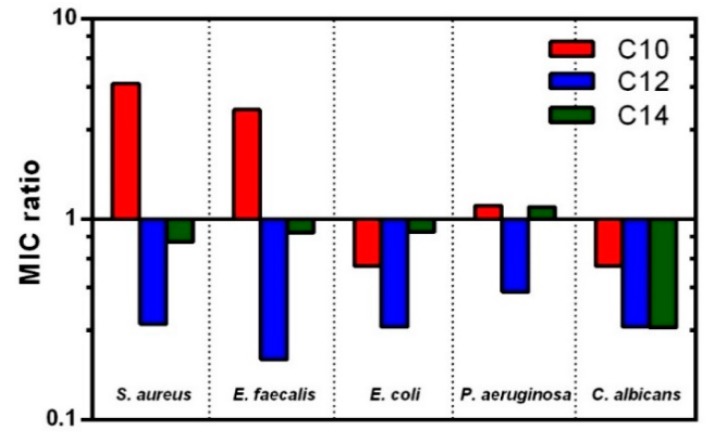
Comparison between MIC values from leucine-based quaternary ammonium surfactants (C10, C12 and C14) without or in the presence of the benzyl group. The MIC ratio is calculated from the ratio of MIC values for leucine surfactants without and with the benzyl group. MICs for surfactants without the benzyl group are from [14].

A comparison between MICs of leucine-based quaternary ammonium surfactants with and without (values are reported in [14]) the *N*-benzyl group may indicate the contribution of this substituent to the antimicrobial activity. Overall, the antimicrobial activity is negatively affected by the introduction of the *N*-benzyl group, as is evident for the C12 and C14 derivatives. Conversely, an enhanced effect by the presence of the benzyl group was observed in the case of the C10 derivative against the two Gram-positive bacterial species *Staphylococcus aureus* and *Enterococcus faecalis* (Figure 5).

The different effect exerted by the introduction of the *N*-benzyl group also affects the selectivity index (EC_50_/MIC), which can be considered as a parameter indicating the relationship between the effectiveness and safety of the surfactants. Indeed, selectivity indexes higher than 10 were calculated only for C10 LEU BENZ against the two tested Gram-positive bacteria, indicating a possible selective antimicrobial effect at non-cytotoxic concentrations (Table S2).

## 4. Discussion

Research regarding surfactants is increasingly focused on the synthesis and discovery of amphiphilic molecules showing good surface adsorption properties, together with additional biological activities. Among these, one of the most explored is the antimicrobial activity, which is characteristic of cationic surfactants, including quaternary ammonium compounds [19,20]. Indeed, quaternary ammonium amphiphiles are well-known for their antibacterial (especially toward Gram-positive) and antifungal activities, which is why they are available as marketed products for a wide range of applications, including pharmaceuticals and cosmetics. Different modifications have been proposed to enhance the antimicrobial activity of these amphiphiles, including addition of alkyl or benzyl groups and different aromatic moieties [21,22]. The modification by the addition of *N*-benzyl groups is quite common and these surfactants are present in many of the commercial products (e.g., benzalkonium chloride) as disinfectants or biocides [23].

In terms of adsorption properties and lowering of surface tension, the substitution of a methyl group with a benzyl group exerts a double effect by increasing the hydrophobicity and the steric hindrance of the ammonium quaternary polar head. Indeed, leucine-based surfactants in the presence of the *N*-benzyl group have CMC values lower than the corresponding *N*-methylated compounds due to the higher hydrophobicity. Moreover, only C10 *N*-benzyl surfactants maintained γ_CMC_ values around 30 mN/m, similarly to all *N*-methylated compounds, while, for the C12 and C14 *N*-benzyl derivatives, the γ_CMC_ values increased up to 40 mN/m, probably due to the combined effect of a larger size of the polar head and the longer hydrophobic tail.

Cytotoxicity studies with these materials were performed on two cell lines relevant to the oral (Caco-2) and respiratory (Calu-3) route of administration. The data showed a clear link between the EC_50_ values and CMC, similarly to *N*-methylated compounds and other classes of surfactants [6,24,25]. Slightly lower EC_50_ values were calculated for the C12 and C14 derivatives of *N*-benzyl surfactants, while, for the C10 derivatives, these values were comparable to the counterpart *N*-methylated compound. A clear relationship between EC_50_ and the other surface parameters (*Γ*_max_ and *A*_min_) was not found, suggesting that CMC is the most sensitive physicochemical property affecting cytotoxicity.

The synthesised surfactants containing the *N*-benzyl group displayed a wide range of antimicrobial activity against Gram-positive and Gram-negative bacteria, as well as fungi. Despite the large number of modifications performed on the quaternary ammonium group reported in the literature for antimicrobials, it is not straightforward to perform a direct comparison between the antimicrobial activity of the same compound before and after the addition of a benzyl group. It is known that the antimicrobial activity of quaternary ammonium salts depends on the irreversible binding to the phospholipids and proteins of the membrane, thereby impairing permeability [19,26]. Specifically, the different nature of the *N*-substituent affects the hydrophobicity of the compounds and their ability to adsorb onto the micro-organism surface, as well as the ability of the molecules to be inserted inside the membrane. For instance, with isatin, an indole derivative, the introduction of a benzyl group decreased the activity against Gram-negative bacteria and enhanced the activity against Gram-positive bacteria [27]. On the other hand, the more lipophilic imidazolium salts, in which the quaternary ammonium is directly linked to a benzyl group, showed a lower antimicrobial activity than the more hydrophilic analogs [28]. In the case of the synthesised leucine-based surfactant series, the introduction of the benzyl group has exerted an opposite effect in terms of antimicrobial activity. An enhanced inhibition of growth was observed only for the C10 derivative against Gram-positive bacteria, while in the other cases (the C12 and C14 derivatives as well as C10 against the Gram-negatives and *C. albicans*), there was a decrease in activity since higher MIC values were obtained. This result can be explained by the increased hydrophobicity of the compounds leading to an effective insertion of the surfactant inside the membrane in the case of the shortest derivative (C10).

## 5. Conclusions

The synthesised leucine-based quaternary ammonium surfactants *N*-functionalised with a benzyl group showed good surface properties and a toxicological profile comparable to BAC as a reference compound. Moreover, they maintained the wide antimicrobial spectrum of activity of all quaternary ammonium compounds, being highly effective in terms of MIC toward the Gram-positive bacteria. The results also highlighted the different effects exerted by the presence of the *N*-benzyl group on the selectivity among the different tested micro-organisms and in relation to the different length of the hydrocarbon chain of the amphiphiles. Indeed, the antimicrobial activity was enhanced only for the *N*-benzyl C10 derivative toward Gram-positive bacteria, which may be related to an effective adsorption of this surfactant and insertion of its hydrophobic chain inside the bacterial membrane.

## Figures and Tables

**Table 1:** A list of the strains that were used to test the antimicrobial activity of the synthesised quaternary ammonium surfactants.

Species	Strain	Note
*Staphylococcus aureus*	ATCC 25923	Gram-positive
*Enterococcus faecalis*	ATCC 29212	Gram-positive
*Escherichia coli*	ATCC 25922	Gram-negative
*Pseudomonas aeruginosa*	ATCC 27853	Gram-negative
*Candida albicans*	ATCC 24433	Fungus, Yeast

**Table 2:** Surface tension parameters and critical micelle concentration (CMC) for the synthesised quaternary ammonium leucine surfactants (C10 LEU BENZ, C12 LEU BENZ and C14 LEU BENZ) in comparison to BAC as determined by tensiometric measurements. Values are the mean ± SD of three replicates.

Surfactant	Tensiometry			
	CMC (mM)	γ_CMC_ (mN/m)	10^6^ *Γ*_max_ (mol/m^2^)	*A*_min_ (A^2^)
BAC	1.02 ± 0.06	31.16 ± 0.05	5.35 ± 0.27	31.08 ± 1.60
C10 LEU BENZ	1.88 ± 0.16	31.75 ± 0.09	6.76 ± 0.22	24.09 ± 1.45
C12 LEU BENZ	0.92 ± 0.11	40.38 ± 0.13	6.10 ± 0.24	27.27 ± 1.34
C14 LEU BENZ	0.12 ± 0.08	40.35 ± 0.11	7.39 ± 0.31	22.46 ± 1.11

**Table 3:** Cytotoxicity parameters (EC_50_) and HC_50_ values of the synthesised quaternary ammonium leucine surfactants (C10 LEU BENZ, C12 LEU BENZ and C14 LEU BENZ) in comparison to BAC as determined by MTS, LDH and haemolytic assays. EC_50_ is the concentration of surfactants that caused a 50% reduction of viable cells (MTT assay) or a 50% release of LDH (LDH assay). HC_50_ is the concentration of surfactants that caused a 50% haemoglobin release from erythrocytes. Results are mean ± SD of at least three independent repeated experiments.

Surfactant	MTS Assay (EC_50_ mM)		LDH Assay (EC_50_ mM)		Haemolysis (HC_50_ mM)
	Caco-2	Calu-3	Caco-2	Calu-3	
BAC	0.033 ± 0.001	0.022 ± 0.005	0.048 ± 0.004	0.029 ± 0.005	0.064 ± 0.004
C10 LEU BENZ	0.160 ± 0.007	0.117 ± 0.005	0.158 ± 0.015	0.187 ± 0.024	0.148 ± 0.018
C12 LEU BENZ	0.016 ± 0.003	0.011 ± 0.002	0.019 ± 0.002	0.019 ± 0.003	0.054 ± 0.004
C14 LEU BENZ	0.013 ± 0.002	0.014 ± 0.002	0.012 ± 0.002	0.014 ± 0.002	0.027 ± 0.006

**Table 4:** Calculated minimum inhibitory concentration (MIC) values (mg/L and μM) for the synthesised quaternary ammonium leucine surfactants (C10 LEU BENZ, C12 LEU BENZ and C14 LEU BENZ) and the reference compound BAC.

Surfactant	MIC Values									
	*S. aureus*		*E. faecalis*		*E. coli*		*P. aeruginosa*		*C. albicans*	
	mg/L	µM	mg/L	µM	mg/L	µM	mg/L	µM	mg/L	µM
BAC	1	2.9	1	2.9	4	11.5	32	91.8	4	11.5
C10 LEU BENZ	2	3.8	4	7.7	64	123.7	>128	>247.5	64	123.7
C12 LEU BENZ	4–8	10.6	4–8	10.6	32	58.6	128	234.6	16	29.3
C14 LEU BENZ	1–2	2.6	2	3.5	16	27.9	128	223.1	8	13.9

## Supplementary Materials

The following are available online at https://www.mdpi.com/1999-4923/11/6/287/s1, Table S1: Chemical structures and H^1^-NMR interpretation for benzyl quaternary ammonium leucine-based surfactants. Table S2: Selectivity index (EC_50_/MIC) for the synthesised leucine-based quaternary ammonium surfactants in comparison to BAC. EC_50_ values are from the MTS assay.
