# Supplementary material for: Quaternary Ammonium Leucine-Based Surfactants: The Effect of a Benzyl Group on Physicochemical Properties and Antimicrobial Activity
Source: Pharmaceutics. 2019 Jun 19;11(6):287. doi: 10.3390/pharmaceutics11060287 (PMC6631462; doi:10.3390/pharmaceutics11060287)
Supplement: Supplementary file 1 [file pharmaceutics-11-00287-s001.pdf]

pharmaceutics

MDPI

Supplementary materials: Quaternary Ammonium Leucine-Based Surfactants: The Effect of a Benzyl Group on Physicochemical Properties and Antimicrobial Activity

Diego Romano Perinelli, Dezemona Petrelli, Luca Agostino Vitali, Giulia Bonacucina, Marco Cespi, Driton Vllasaliu, Gianfabio Giorgioni and Giovanni Filippo Palmieri

Table S1. Chemical structures and H^1-NMR interpretation for benzyl quaternary ammonium leucine-based surfactants.

**C10 LEU BENZ**	^{1}H NMR (DMSO) δ 7.68-7.50 (m, 5H, ArH); 4.83-4.60 (dd, 2H, CH_2); 4.39-4.20 (m, 3H, CH and CH_2); 3.03-3.01 (m, 8H, CH_3 and CH_2); 2.15-1.95 (m, 2H, CH_2); 1.66-1.60 (m, 2H, CH_2); 1.50-1.44 (m, 1H, CH); 1.36-1.20 (m, 12H, CH_2); 1.02-0.94 (m, 6H, CH_3); 0.85 (t, 3H, CH_3).
**C12 LEU BENZ**	^{1}H NMR (DMSO) δ 7.68-7.50 (m, 5H, ArH); 4.83-4.56 (dd, 2H, CH_2); 4.39-4.20 (m, 3H, CH and CH_2); 3.18 (s, 6H, CH_3); 3.06-3.01 (m, 2H, CH_2); 1.90-1.81 (m, 2H, CH_2); 1.66-1.60 (m, 2H, CH_2); 1.48-1.44 (m, 1H, CH); 1.28-1.20 (m, 16H, CH_2); 1.02-0.94 (m, 6H, CH_3); 0.85 (t, 3H, CH_3).
**C14 LEU BENZ**	^{1}H NMR (DMSO) δ 7.68-7.50 (m, 5H, ArH); 4.83-4.56 (dd, 2H, CH_2); 4.38-4.29 (m, 3H, CH and CH_2); 3.18 (s, 6H, CH_3); 3.06-2.97 (m, 2H, CH_2); 1.89-1.81 (m, 2H, CH_2); 1.70-1.59 (m, 2H, CH_2); 1.48-1.40 (m, 1H, CH); 1.38-1.12 (m, 20H, CH_2); 1.04-0.83 (m, 9H, CH_3).

Table S2. Selectivity index (EC50/MIC) for the synthesized leucine-based quaternary ammonium surfactants in comparison to BAC. EC50 values are from MTS assay.

	**Selectivity index EC_{50}/MIC**				
	**Caco-2**				
	S. aureus	***E. faecalis***	***E. coli***	***P. aeruginosa***	***C. albicans***
BAC	11.5	11.5	2.9	0.4	2.9
C10 LEU BENZ	42.1	28.1	1.3	0.6	1.3
C12 LEU BENZ	1.5	1.5	0.3	0.07	0.5
C14 LEU BENZ	5	3.7	0.5	0.06	0.9
	**Calu-3**				
BAC	7.7	7.7	1.9	0.2	1.9
C10 LEU BENZ	30.8	15.2	0.9	0.5	0.9
C12 LEU BENZ	1.0	1.0	0.2	0.05	0.4
C14 LEU BENZ	5.4	4.0	0.5	0.06	1.0
